# The Role of β-Defensin 1 Against Porphyromonas gingivalis Lipopolysaccharide-Mediated Inflammation in the THP-1 Cell Line

**DOI:** 10.7759/cureus.50880

**Published:** 2023-12-21

**Authors:** Harini Venkata Subbiah, Polani Ramesh Babu, Usha Subbiah

**Affiliations:** 1 Human Genetics Research Centre, Sree Balaji Dental College & Hospital, Bharath Institute of Higher Education and Research, Chennai, IND; 2 Centre for Materials Engineering and Regenerative Medicine, Bharath Institute of Higher Education and Research, Chennai, IND

**Keywords:** thp-1, β-defensin 1, lipopolysaccharide, porphyromonas gingivalis, periodontitis

## Abstract

Introduction

*Porphyromonas gingivalis* lipopolysaccharide (Pg-LPS) is one of the crucial virulence factors of periodontitis. Antimicrobial peptides (AMPs) are emerging as alternatives or adjuncts to antibiotics in the treatment of microbial infections. In this study, cytotoxicity, anti-inflammatory activity, anti-oxidative stress, cell cycle analysis, and apoptosis properties of AMP, β-defensin 1, were studied in Pg-LPS-stimulated THP-1 (Tohoku Hospital Pediatrics - 1) cell line.

Methods

The cytotoxic nature of Pg-LPS and β-defensin 1 was studied by the MTT (3-(4,5-dimethylthiazol-2-yl)-2,5-diphenyltetrazolium bromide) method. The cytotoxic effect of β-defensin 1 on Pg-LPS-stimulated THP-1 cells was also studied by the same method. The anti-inflammatory role of β-defensin 1 against cyclooxygenase (COX), lipoxygenase (LOX), myeloperoxidase (MPO), and inducible nitric oxide synthase activities were studied. The anti-oxidative nature of β-defensin 1 was analyzed by measuring reactive oxygen species (ROS) generation by dichlorodihydrofluorescein diacetate (DCFDA) assay. Cell cycle distribution and apoptosis were studied by flow cytometry. The hemolytic nature of β-defensin 1 was predicted using the HemoPred web tool.

Results

The results of the study demonstrated that Pg-LPS showed dose-dependent cytotoxicity to THP-1 cells. β-Defensin 1 had dose-dependent cytotoxicity to THP-1 cells and showed a protective effect on THP-cells up to 1 µg/mL of Pg-LPS, beyond which cell viability decreased. β-Defensin 1 inhibited COX, LOX, MPO, and inducible nitric oxide synthase activities in a concentration-dependent manner. β-Defensin 1 showed anti-oxidative activity by suppressing the generation of ROS measured through fluorescence intensity. From the cell cycle analysis, it was found that β-defensin 1 was able to reduce the Pg-LPS-induced cell cycle arrest at the G0/G1 phase. From the apoptosis profile, β-defensin 1 was found to increase the live cells when compared to THP-1 cells stimulated only with Pg-LPS, indicating that β-defensin 1 provided a protective role to THP-1 cells. β-Defensin 1 was found to be hemolytic in nature by the HemoPred web tool.

Conclusion

β-Defensin 1 exerted multifunctional activities and can be considered a promising agent for controlling periodontitis.

## Introduction

Periodontitis is driven by a dysbiotic microbiome and inflammation mediated by the host, resulting in dysfunction and damage to periodontal tissues [1]. The primary function of the periodontium is to support and anchor the teeth within the jaw. Periodontal dysfunction is characterized by periodontal breakdown, indicated by increased pocket depth, furcation involvement, bone loss, tooth hypermobility, and masticatory dysfunction. "Porphyromonas gingivalis, Tannerella forsythia, and Treponema denticola," belonging to the red complex species, are significant periodontopathogenic bacteria. They trigger inflammation of the gingiva and progressively affect and destroy the periodontal ligament and adjacent supporting alveolar bone, leading to tooth loss [2]. Porphyromonas gingivalis (Pg) is a keystone anaerobic gram-negative bacterium in periodontitis, with virulence factors such as lipopolysaccharide (Pg-LPS), gingipains, hemagglutinins, and fimbriae.

Pg-LPS is a "pathogen-associated molecular pattern" (PAMP) recognized by toll-like receptors (TLR, "pattern recognition receptors") expressed on various immune cells, including macrophages and dendritic cells. This interaction leads to the synthesis of immune-inflammatory molecules like chemokines and cytokines, which resolve bacterial infections. When the infection is not resolved, more molecules and cells of the innate and adaptive immune systems are activated, leading to pathological chronic inflammation that can cause damage to the periodontium [3]. Pg-LPS interacts with TLR4 or TLR2, but the TLR2 activity of Pg-LPS is attributed to the presence of conjugated lipoprotein [4].

Antimicrobial peptides (AMPs) are important constituents of innate immune responses and offer initial defense against bacterial infections [5]. β-Defensins, AMPs produced by epithelial cells in organs like the skin, respiratory tract, oral cavity, gastrointestinal tract, and genitourinary tract, have a broad range of antimicrobial activity against gram-positive and gram-negative bacteria, fungi, and viruses [6]. These cationic, amphipathic molecules destroy bacteria by targeting negatively charged phospholipids of bacterial membranes, creating pores that lead to osmotic lysis [7]. Additionally, they link innate and adaptive immunity by interacting with the CCR6 receptor on immature dendritic cells and T-cells, recruiting these cells to the site of infection [8]. The β-defensin family, consisting of six members (β-defensins 1-6), includes β-defensin 1, which is constitutively expressed and plays a crucial role in preventing natural flora from becoming opportunistic pathogens [6]. β-Defensin 1 is especially important for preventing infections at mucosal surfaces, which are common entry points for pathogens, providing rapid protection before the adaptive immune system responds. It is also implicated in wound healing, aiding in tissue repair by promoting cell migration and proliferation. Given its antimicrobial and immunomodulatory properties, β-defensin 1 is being researched for use in strategies to combat infectious diseases.

AMPs have various advantages, including low toxicity to eukaryotic cells, good thermal stability and water solubility, low molecular weight, and synergistic effects with other antibiotics in neutralizing endotoxins [9]. While some resistance mechanisms like upregulation of efflux pumps, membrane modifications, proteolytic cleavage of peptides, and LPS alteration have been reported, AMPs are still considered potential therapeutic agents, particularly when used in appropriate concentrations and in combination with other antimicrobial agents to fight microbial infections [10].

THP-1 (Tohoku Hospital Pediatrics - 1) is a human monocyte leukemia cell line that differentiates into macrophage-like cells when treated with phorbol esters. These differentiated THP-1 cells are similar to native monocyte-derived macrophages. In this study, the effects of β-defensin 1 on cell viability, anti-inflammation, reactive oxygen species (ROS) generation, cell cycle progression, and apoptosis in Pg-LPS stimulated THP-1 cells were examined.

## Materials and methods

Chemicals

β-Defensin 1 peptide was purchased from ThermoFisher Scientific, USA. Standard Pg-LPS was procured from Invivogen, USA. The THP-1 cell line was purchased from the National Centre for Cell Science (NCCS), Pune, India, and was maintained in Dulbecco's Modified Eagle's Medium (DMEM, Sigma Aldrich, USA).

Cells seeding in a 96-well plate

The THP-1 cell line was cultured in DMEM and supplemented with 10% FBS, sodium bicarbonate, L-glutamine, and an antibiotic solution containing penicillin (100 U/mL), amphotericin B (2.5 µg/mL), and streptomycin (100 µg/mL). The cells were maintained in a 5% humidified CO_2_ incubator (NBS Eppendorf, Germany) at 37°C. To induce differentiation into macrophages, the THP-1 cell line was treated with 25 ng/mL phorbol 12-myristate 13-acetate (PMA) (cat. 91585, Sigma-Aldrich, USA) and incubated for 48 hours. Following incubation, the cells were seeded in a 96-well culture plate at a density of 5x10^4^ cells/well and incubated for 24 hours in a humidified 5% CO_2_ incubator at 37°C.

Cytotoxicity evaluation of Pg-LPS

After incubation of 24 hours, triplicate additions of Pg-LPS in various concentrations (0.25 µg, 0.5 µg, 1 µg, 2 µg, and 4 µg/mL) were done appropriately. The cells were then incubated for 24 hours at 37ºC in an incubator with a humidity level of 5% CO_2_. Also “Non-treated control cells” were maintained. The inhibitory concentration (IC50) value of Pg-LPS was determined by the ED50 software.

Inverted phase contrast microscopy (IPCM) using an Olympus CKX41 microscope (Olympus, Japan) equipped with an Optika Pro5 CCD camera (Optika, Italy) was utilized to directly observe the cells. Cell viability was determined using the MTT (3-(4,5-Dimethylthiazol-2-yl)-2,5-diphenyltetrazolium bromide) assay (Sigma, M-5655). The MTT assay is a colorimetric method for measuring cellular metabolic activity. The positively charged and lipophilic MTT reagent can permeate the cell and mitochondrial membranes of viable cells and is reduced to formazan by metabolically active cells. This process is based on the ability of nicotinamide adenine dinucleotide phosphate (NADPH)-dependent cellular oxidoreductase enzymes to reduce the tetrazolium dye MTT to its insoluble formazan, which is purple in color. These NAD(P)H-dependent enzymes reflect the number of viable cells present. A solubilization solution is added to dissolve the insoluble purple formazan product into a colored solution. The absorbance of this colored solution can be quantified by measuring at approximately 570 nm using a spectrophotometer.

Cytotoxicity assay by the MTT method

The whole plate was analyzed under a microscope with an IPCM, and the results were photographed. Rounding or shrinking of cells, granulation, and vacuolization of the cytoplasm were considered signs of cytotoxicity. After incubation for 24 hours, MTT reagent was added and incubated for 4 hours at 37ºC. The purple formazan crystals were dissolved in dimethyl sulfoxide (DMSO) and the absorbance (OD) was measured at 570 nm using a microplate reader. The percentage viability was computed by the following formula

$Percentage\: viability\: = \: (Mean \: OD \: of\: samples \times 100)/(Mean\: OD \: of\: control\: group)$

Cytotoxicity evaluation of β-defensin 1

For the evaluation of β-defensin 1 toxicity, after differentiating THP-1 cells into macrophages and incubating them for 24 hours, freshly prepared β-defensin 1 in 5% DMEM was serially diluted through a two-fold process (200 ng, 100 ng, 50 ng, 25 ng, and 12.5 ng/mL in DMEM). The diluted samples were then added in triplicates to the corresponding wells and incubated for another 24 hours at 37°C in a humidified 5% CO2 incubator. "Non-treated control cells" were also maintained under similar conditions. The cells were observed under an IPCM, and their vitality was assessed using the MTT assay as described earlier. The IC50 value of β-defensin 1 was calculated using the ED50 software.

Cytotoxic evaluation of β-defensin 1 against Pg-LPS-treated THP-1 cells

To know the effect of β-defensin 1 on Pg-LPS treated THP-1 cell line, after differentiation of THP-1 cells into macrophages and 24 hours of incubation, different concentrations of Pg-LPS (0.25µg, 0.5 µg, 1 µg, 2 µg, and 4 µg/mL in DMEM) were added in triplicates to the corresponding wells for 1 hour and IC50 concentration of β-defensin 1 was added and incubated for 24 hours at 37ºC in a humidified 5% CO2 incubator. “Non-treated control cells” were also maintained. The viability of cells was studied by direct observation of cells by an IPCM and followed by MTT assay as mentioned above.

Study of the anti-inflammatory role of β-defensin 1

For inducing differentiation of THP-1 cells into macrophages, the cell line was premixed with 25 ng/mL PMA and incubated for 48 hours. After incubation, 5x10^4^ cells were activated with Pg-LPS (LPS: 1 µg/mL) for one hour. Pg-LPS stimulated cells were exposed to different concentrations (6.25, 12.5, and 25 ng/mL) of β-defensin 1 and incubated for 24 hours. After incubation, the following anti-inflammatory assays were conducted utilizing the cell lysate.

Cyclooxygenase Activity

The cyclooxygenase (COX) activity was assayed by the method of Walker and Gierse [11]. The cell lysate was incubated for one minute at 25°C with Tris-HCl buffer (pH 8), glutathione 5 mM/L, and hemoglobin 5 mM/L. Arachidonic acid 200 mM/L was added to initiate the reaction, and after 20 minutes at 37°C, 200 µL of 10% trichloroacetic acid in 1 N hydrochloric acid was added to terminate the reaction. After centrifugation and addition of 200 µL of 1% thiobarbituric acid, the tubes were boiled for 20 minutes. After cooling, the tubes were centrifuged for three minutes. By reading absorbance at 632 nm, COX activity was measured, and the percentage of COX activity inhibition was calculated as per the following formula

$Percentage\: inhibition\: =\: (Absorbance \: of \: control\: -\: Absorbance\: of \: test) \times 100/(Absorbance \: of \: control)$

Lipoxygenase Activity

The lipoxygenase (LOX) activity was determined using the method described by Axelrod in 1981 [12]. The reaction mixture consisted of 50 µL of cell lysate, Tris-HCl buffer (pH 7.4), and 200 µL of sodium linoleate. By determining absorbance at 234 nm (Agilent Cary 60), the formation of 5-hydroxyeicosatetraenoic acid, an indicator of LOX activity, was tracked. The percentage inhibition of the enzyme was computed as

$Percentage\: inhibition\: =\: (Absorbance \: of \: control\: -\: Absorbance\: of \: test) \times 100/(Absorbance \: of \: control)$

Myeloperoxidase Activity

Cell lysate was homogenized in hexadecyltrimethyl ammonium bromide (HTAB) and potassium phosphate buffer [13]. The samples were centrifuged at 2000*g* for 30 minutes at 4°C and the activity of myeloperoxidase (MPO) enzyme was measured with the supernatant. MPO in the sample was stimulated by adding 50 mM phosphate buffer (pH 6) containing 1.67 mg/mL guaiacol and 0.0005% hydrogen peroxide. The absorbance change at 460 nm was determined. The MPO activity (U) was shown as units per milliliter of cell lysate. At 25 °C, one unit of MPO activity corresponds to the degradation of 1µM peroxide per minute.

$U = (&Delta;OD \times 4 \times Vt \times Dilution factor) / (L \times &pound;470 \times &Delta;t \times Vs)$

where ΔOD is the density change, Vt is the total volume (mL) (1.1 mL ), L is the light path (1 cm), and £470 is the extinction coefficient for tetraguaiacol (26.6 mM^-1^·cm^-1^).

Inducible Nitric Oxide Synthase

Nitric oxide synthase was measured using the method reported by Salter et al. [14]. The cell lysate was homogenized in 2 ml of 4-(2-hydroxyethyl)-1-piperazineethanesulfonic acid (HEPES) buffer. The assay system contained 0.1 mL of 2 µmol/L L-Arginine, 0.1 mL of 4 µmol/L manganese chloride, 0.1 mL of 10 mmol/L 30 µg dithiothreitol (DTT), 0.1 mL of 1 mmol/L NADPH, 0.1 mL of 4 µmol/L tetrahydropterin, 0.1 mL of 10µmol/L oxygenated hemoglobin, and 0.1 mL of cell lysate. Enzyme activity was measured by recording an increase in absorbance at 401 nm, and the following equation was used:

$Percentage\: inhibition\: =\: (Absorbance \: of \: control\: -\: Absorbance\: of \: test) \times 100/(Absorbance \: of \: control)$

Analysis of intracellular reactive oxygen species (ROS) generation using a fluorescent microscope

Differentiated THP-1 cells were treated with Pg-LPS (LPS: 1 µg/mL) for one hour, and β-defensin 1 at its IC50 concentration (22.62 ng/mL) as determined by the COX inhibition assay was added and incubated for 24 hours. Untreated control and Pg-LPS treated wells were also maintained. The cells were washed with PBS, and 50 µl of Dichlorodihydrofluorescein diacetate (DCFDA) was added, followed by incubation for 30 minutes. After incubation, excess dye was removed with PBS, and fluorescence was observed using a fluorescent microscope (Olympus CKX41 with Optika Pro5 CCD camera) and quantified with a fluorimeter at 470 nm excitation and emission at 635 nm (Qubit 3.0, Life Technologies, USA), displaying the results in arbitrary units (AU).

Analysis of cell cycle distribution by flow cytometry

Differentiated THP-1 cells were stimulated with Pg-LPS (LPS: 1 µg/mL) for one hour, and β-defensin 1 at its IC50 concentration (22.62 ng/mL), as per the COX inhibition assay, was added and incubated for 24 hours. Untreated control and Pg-LPS stimulated cells were also maintained. The MUSE Cell Cycle Kit was added, and the manufacturer's instructions were followed.

Analysis of apoptosis by flow cytometry

Differentiated THP-1 cells were treated with Pg-LPS (LPS: 1 µg/mL) for 1 hour and β-defensin 1 with its IC50 concentration (22.62 ng/mL) as per COX inhibition assay was added and incubated for 24 hours. Untreated control and Pg-LPS treated wells were also maintained. To the tubes, 100 μL of the Muse™ Annexin V & Dead Cell Reagents were added separately. The tubes were completely mixed by pipetting up and down or spinning at a medium speed for three to five seconds, followed by 20 minutes of incubation in the dark at room temperature. The cells were examined with a Flow Cytometer, gated against untreated control cells, and evaluated for apoptosis with the Muse FCS 3.0 software.

Identification of hemolytic property of β-defensin 1

Undesirable toxicity of AMPs can arise from their hemolytic property toward eukaryotic cells. The hemolytic activity of β-defensin 1 was determined using HemoPred, a web server for predicting the hemolytic activity of peptides (http://codes.bio/hemopred/). HemoPred is a sequence-based predictor that employs a machine learning method, the random forest classifier, and is based on properties such as amino acid composition, dipeptide composition, and physicochemical descriptors [15].

Statistical analysis

Results are represented as mean ± standard deviation (SD). One-way ANOVA and Dunnets test were performed to analyze the data. A p-value of <0.001 was considered statistically significant.

## Results

Effect of Pg-LPS and β-defensin 1 on cell viability

**Figure 1:**
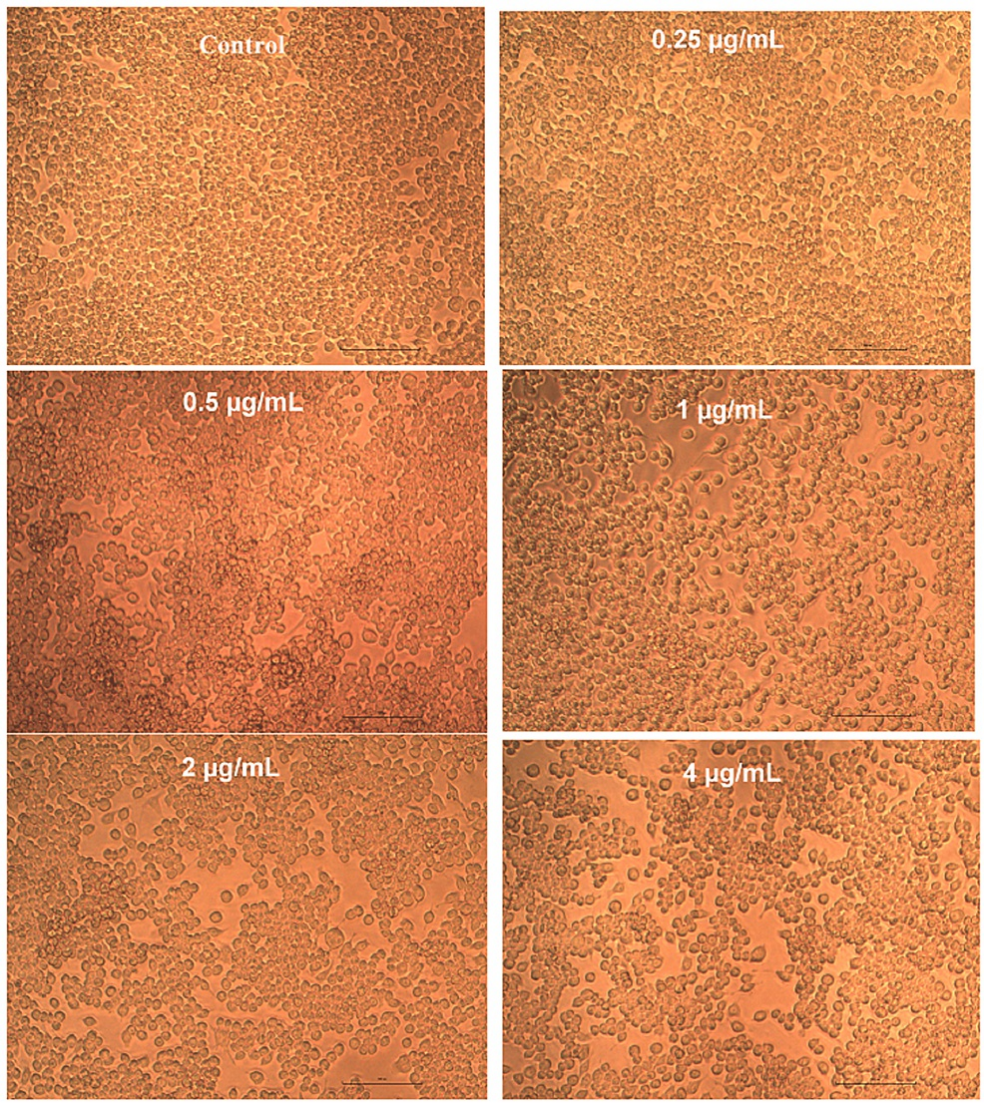
Effect of Pg-LPS on cell viability of THP-1 cells. THP-1 cells were stimulated with different concentrations of Pg-LPS and cell viability was assessed by MTT assay. Pg-LPS: *Porphyromonas gingivalis* lipopolysaccharide, THP-1: Tohoku Hospital Pediatrics - 1, MTT: 3-(4,5-dimethylthiazol-2-yl)-2,5-diphenyltetrazolium bromide.

**Figure 2:**
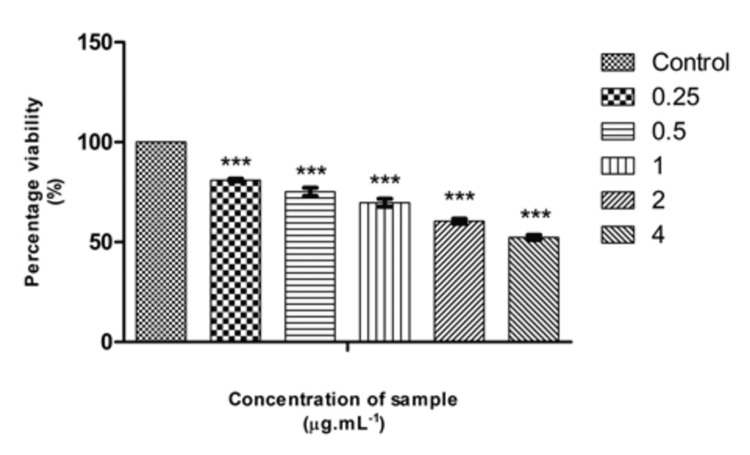
Graphical representation depicting the cytotoxic effect of Pg-LPS by MTT assay. Along the Y-axis, percentage viability is plotted, and along the X-axis, varied concentrations of Pg-LPS are displayed. All experiments were conducted in triplicates, and results are represented as mean ± SD. One-way ANOVA and Dunnett's test were performed to analyze the data. ***p < 0.001 compared to the control group. Pg-LPS: *Porphyromonas gingivalis* lipopolysaccharide, MTT: 3-(4,5-dimethylthiazol-2-yl)-2,5-diphenyltetrazolium bromide.

**Figure 3:**
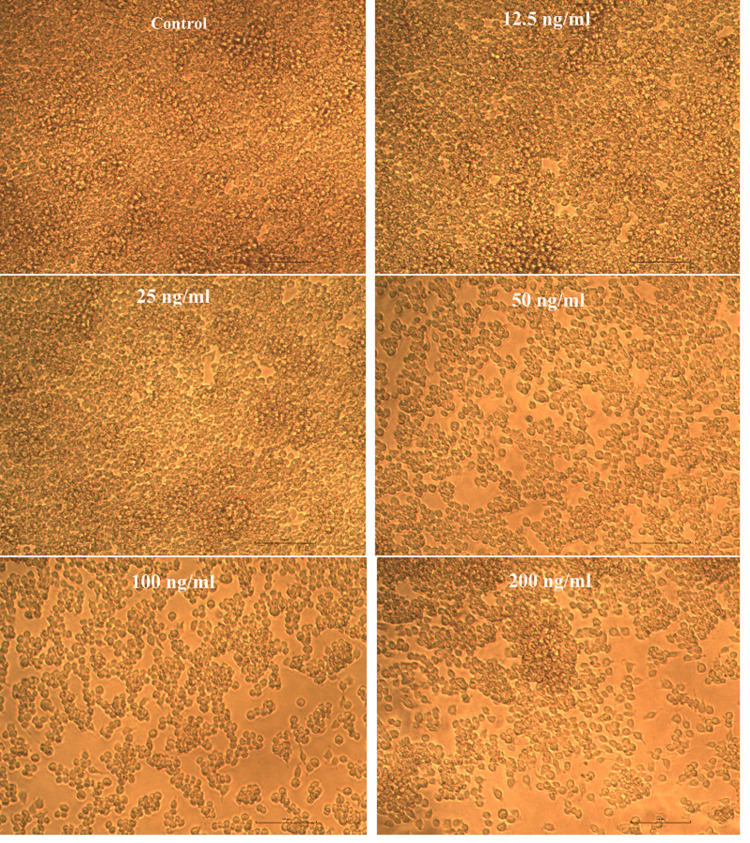
Effect of β-defensin 1 on THP-1 cells. THP-1 cells were treated with different concentrations of β-defensin 1 and cell viability was studied by MTT assay. THP-1: Tohoku Hospital Pediatrics - 1, MTT: 3-(4,5-dimethylthiazol-2-yl)-2,5-diphenyltetrazolium bromide.

**Figure 4:**
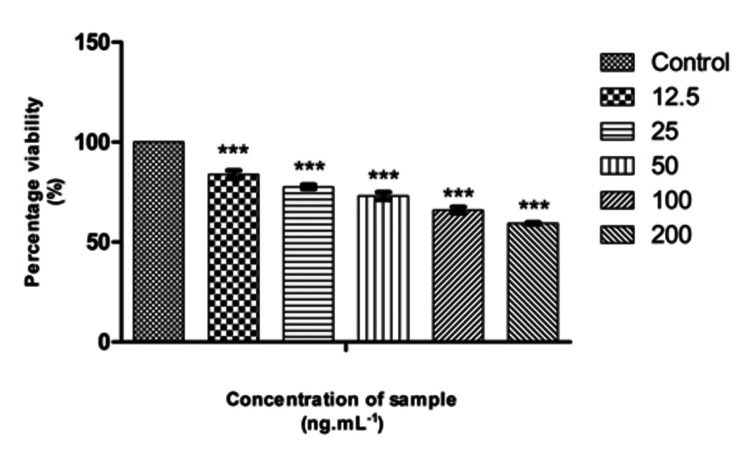
Graphical representation depicting the cytotoxic effect of β-defensin 1 by MTT assay. Along the Y-axis, percentage viability is plotted, and along the X-axis, varied concentrations of β-defensin 1 are displayed. All experiments were conducted in triplicates, and results are represented as mean ± SD. One-way ANOVA and Dunnett's test were performed to analyze the data. ***p < 0.001 compared to the control group. MTT: 3-(4,5-dimethylthiazol-2-yl)-2,5-diphenyltetrazolium bromide.

**Figure 5:**
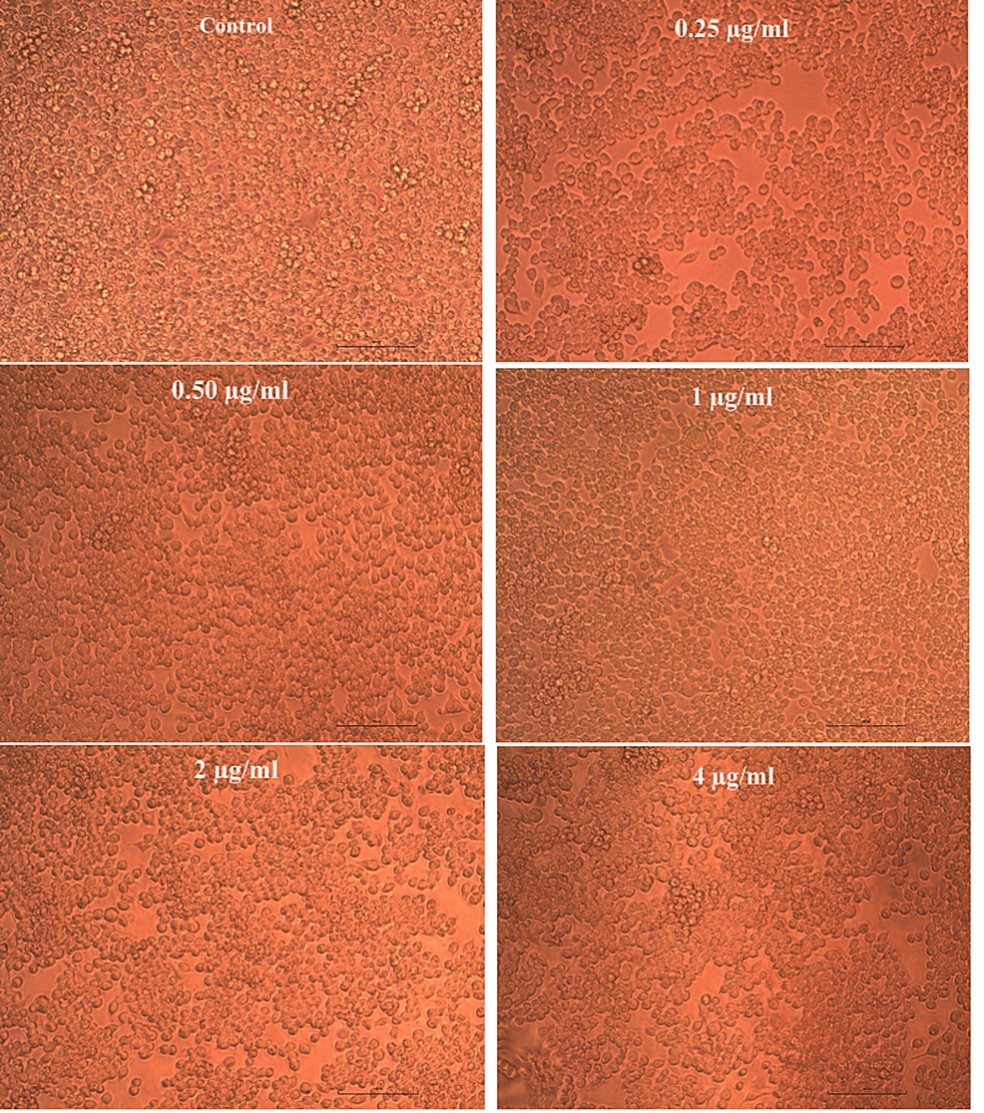
Effect of β-defensin 1 on Pg-LPS stimulated THP-1 cells. Pg-LPS stimulated THP-1 cells were treated with IC50 concentration (260 ng/mL) of β-defensin 1. Pg-LPS: *Porphyromonas gingivalis* lipopolysaccharide, THP-1: Tohoku Hospital Pediatrics - 1.

**Figure 6:**
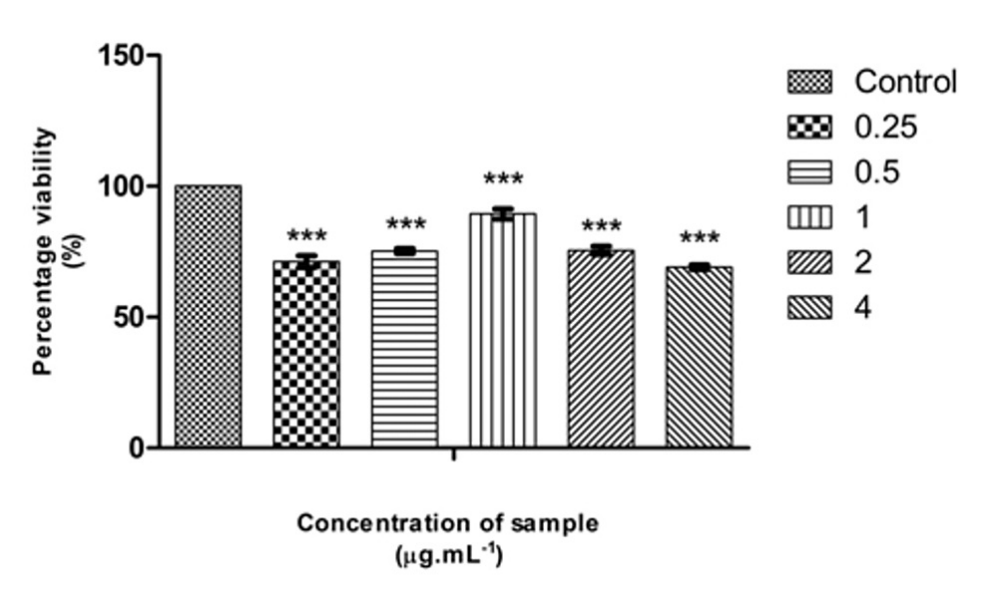
Graphical representation depicting the effect of β-defensin 1 on Pg-LPS stimulated THP-1 cells by MTT assay. Along the Y-axis, percentage viability is plotted, and along the X-axis, varied concentrations of Pg-LPS treated with the IC50 value (260 ng/mL) of β-defensin 1 are displayed. All experiments were conducted in triplicates, and results are represented as mean ± SD. One-way ANOVA and Dunnett's test were performed to analyze the data. ***p < 0.001 compared to the control group. Pg-LPS: *Porphyromonas gingivalis* lipopolysaccharide, THP-1: Tohoku Hospital Pediatrics - 1, MTT: 3-(4,5-dimethylthiazol-2-yl)-2,5-diphenyltetrazolium bromide.

The cytotoxic activity of Pg-LPS and β-defensin 1 was tested against the THP-1 differentiated macrophage cell line. Pg-LPS exhibited dose-dependent cytotoxicity, and the IC50 value was found to be 4.003 µg/mL. β-Defensin 1 also showed dose-dependent cytotoxicity, and its IC50 value was determined to be 260.85 ng/mL. Images illustrating the viability of THP-1 cells after treatment with Pg-LPS and β-defensin 1 are presented in Figures 1, 3. Graphical representations depicting the cytotoxic effects of Pg-LPS and β-defensin 1 are displayed in Figures 2, 4. When the IC50 concentration of β-defensin 1 (260.85 ng/mL) was added to the Pg-LPS stimulated THP-1 cell line, β-defensin 1 increased the viability of THP-1 cells up to a 1 µg/mL concentration of Pg-LPS; beyond that concentration, there was a decrease in viability, as shown in Figures 5, 6. β-Defensin 1 was able to protect THP-1 cells from the cytotoxic effects of Pg-LPS.

Anti-inflammatory role of β-defensin 1 on Pg-LPS stimulated THP-1 differentiated macrophage cell line

To assess the anti-inflammatory role of β-defensin 1, its effect on the inhibition of COX, LOX, MPO, and inducible nitric oxide synthase activities was studied. Pg-LPS stimulated THP-1 cells were treated with different concentrations of β-defensin 1, which showed that the percentage inhibition of COX, LOX, and inducible nitric oxide synthase activities increased in a concentration-dependent manner (Table 1). Additionally, the enzyme activity of MPO decreased in a concentration-dependent manner.

**Table 1:** Percentage inhibition of activities of COX, LOX, MPO, and inducible nitric oxide synthase by different concentrations of β-defensin 1 (6.25, 12.5, and 25 ng/mL) in Pg-LPS stimulated THP-1 cells. The experiment was carried out in duplicates. SD: standard deviation, SE: standard error.

COX	Percentage inhibition 1	Percentage inhibition 2	Average	SD	SE
β-Defensin 1 (ng/mL)					
6.25	19.77	18.4	19.085	0.968736	0.484368145
12.5	36.41	35.09	35.75	0.933381	0.466690476
25	53.21	52.85	53.03	0.254558	0.127279221
LOX	Percentage inhibition 1	Percentage inhibition 2	Average	SD	SE
β-Defensin 1 (ng/mL)					
6.25	21.23	21.74	21.485	0.360624	0.180312229
12.5	38.85	37.31	38.08	1.088944	0.544472222
25	55.2	54.15	54.675	0.742462	0.37123106
MPO	Enzyme activity (U/mL) 1	Enzyme activity (U/mL) 2	Average	SD	SE
β-Defensin 1 (ng/mL)					
6.25	0.0278	0.0281	0.02795	0.000212	0.000106066
12.5	0.0209	0.0211	0.021	0.000141	7.07E-05
25	0.0152	0.0151	0.01515	7.07E-05	3.54E-05
Inducible nitric oxide synthase	Percentage inhibition 1	Percentage inhibition 2	Average	SD	SE
β-Defensin 1 (ng/mL)					
6.25	18.99	19.17	19.08	0.127279	0.042426407
12.5	34.63	33.92	34.275	0.502046	0.167348605
25	50.27	50	50.135	0.190919	0.06363961

Effect of β-defensin 1 on reactive oxygen species generation in THP-1 cells treated with Pg-LPS

**Figure 7:**
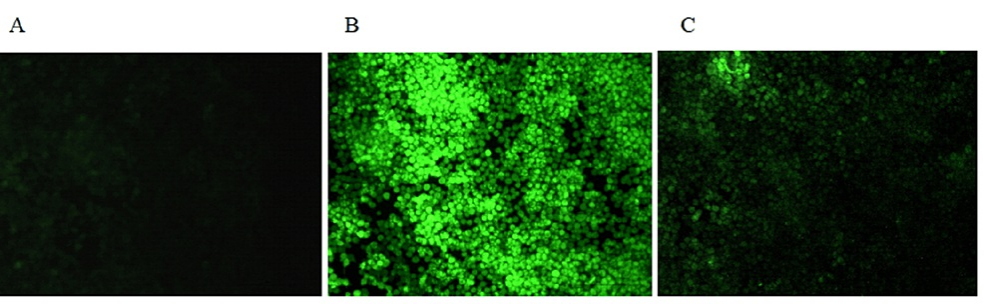
Effect of β-defensin 1 on intracellular ROS generation by Pg-LPS stimulated THP-1 cells. (A) ROS generation by untreated THP-1 cells, (B) ROS generation by Pg-LPS stimulated THP-1 cells, and (C) ROS generation by β-defensin 1 treated Pg-LPS stimulated THP-1 cells. Pg-LPS: *Porphyromonas gingivalis *lipopolysaccharide, THP-1: Tohoku Hospital Pediatrics - 1, ROS: reactive oxygen species.

The effect of β-defensin 1 on ROS generation in THP-1 cells stimulated with Pg-LPS was studied. DCFDA, a fluorogenic dye, measures hydroxyl, peroxyl, and other ROS activities within the cell. After entering the cell, DCFDA is deacetylated by cellular esterases into a non-fluorescent compound. This nonfluorescent chemical is then oxidized by ROS into 2',7'-dichlorofluorescin (DCF), which, upon excitation with a blue filter, emits green fluorescence. When THP-1 cells were stimulated with Pg-LPS, there was a considerable increase in fluorescence, which decreased upon treatment with β-defensin 1, as shown in Figure 7 and Table 2.

**Table 2:** ROS generation as measured by fluorescence intensity in arbitrary units (AU). ROS: reactive oxygen species, Pg-LPS: *Porphyromonas gingivalis* lipopolysaccharide.

Sample	Fluorescence intensity (AU)
Control	2379
Pg-LPS	10743.55
Pg-LPS+ β-defensin 1	5006.24

Cell cycle analysis by flow cytometry

**Figure 8:**
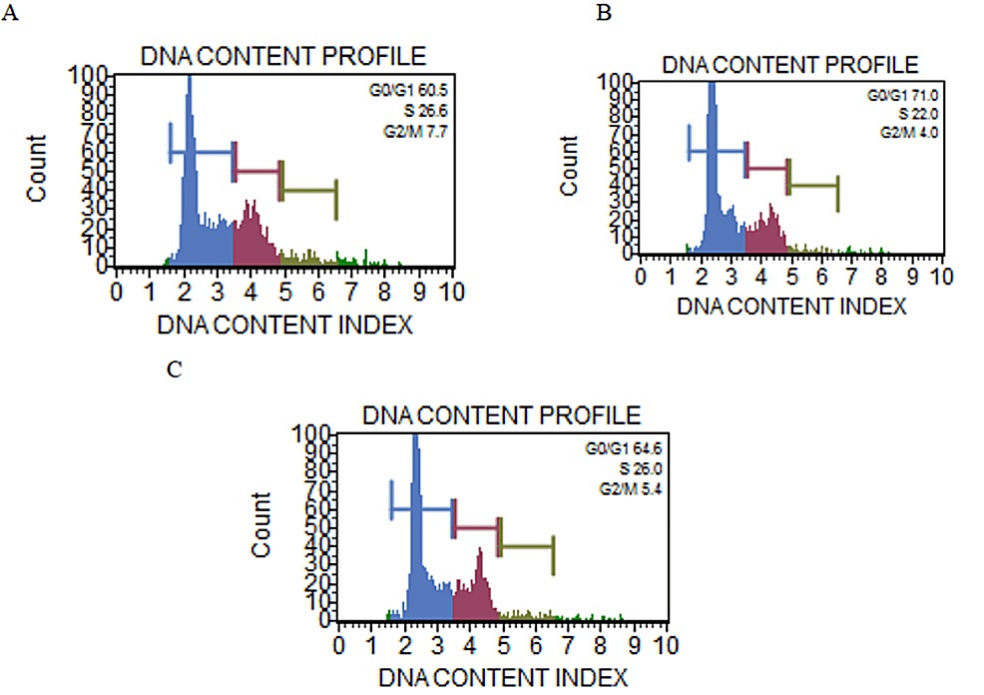
Cell cycle analysis by flow cytometry. (A) DNA content profile of untreated THP-1 cells, (B) DNA content profile of Pg-LPS stimulated THP-1 cells, and (C) DNA content profile of β-defensin 1 treated Pg-LPS stimulated THP-1 cells. Pg-LPS: *Porphyromonas gingivalis* lipopolysaccharide, THP-1: Tohoku Hospital Pediatrics - 1.

The cell cycle progression in THP-1 cells after stimulation with Pg-LPS and also in the presence of β-defensin 1 was investigated. The results showed that Pg-LPS administration produced an increase in the G0/G1 phase of the cell cycle when compared to untreated control cells. Pg-LPS delayed or arrested the transition from the G0/G1 phase of the cell cycle. The arrest in the G0/G1 phase was found to be reduced when co-treated with β-defensin 1. The result is shown in Figure 8. It can be inferred that β-defensin 1 is capable of reducing the Pg-LPS-induced G0/G1 phase arrest.

Detection of apoptosis by flow cytometry

**Figure 9:**
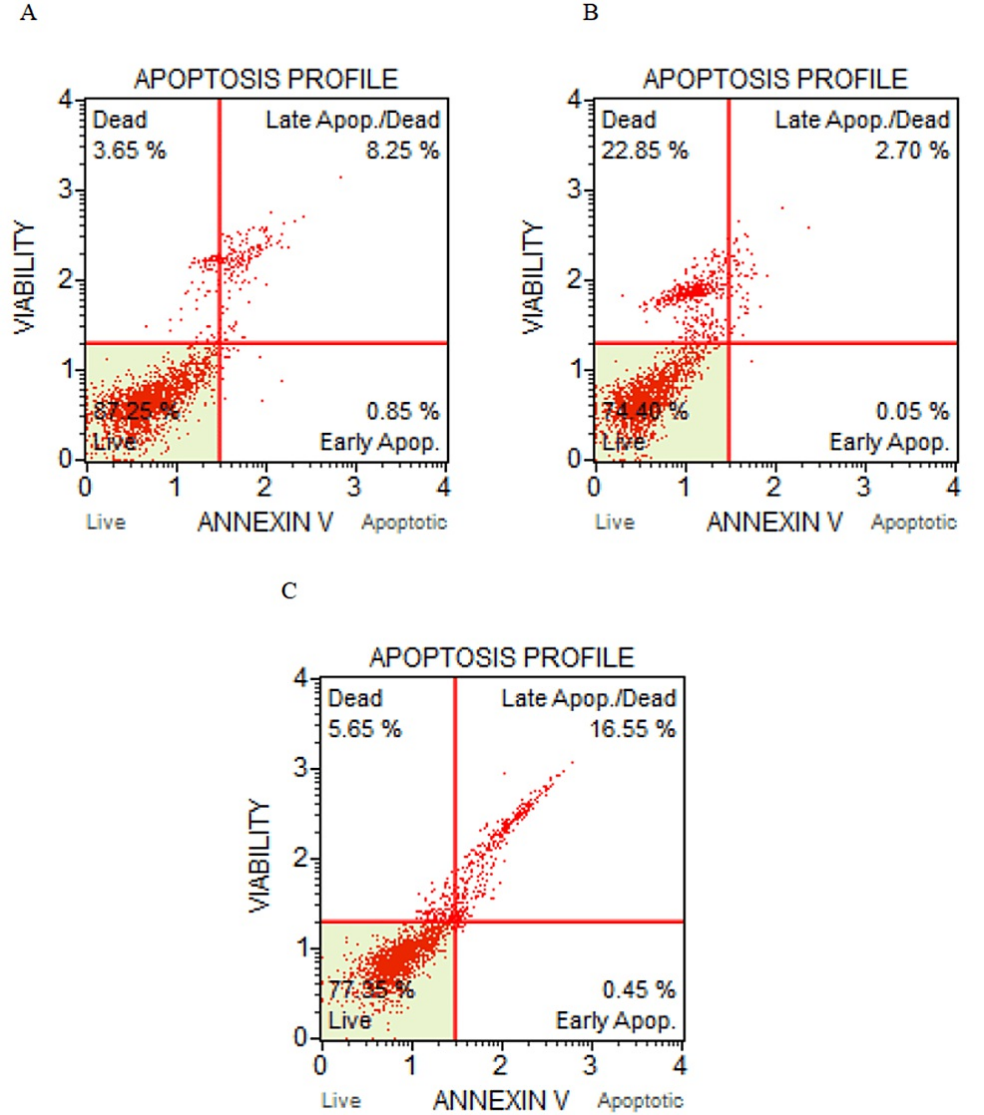
Analysis of apoptosis induction by flow cytometry. (A) Apoptosis profile of untreated THP-1 cells, (B) apoptosis profile of Pg-LPS stimulated THP-1 cells, and (C) apoptosis profile of β-defensin 1 treated Pg-LPS stimulated THP-1 cells. Pg-LPS: *Porphyromonas gingivalis* lipopolysaccharide, THP-1: Tohoku Hospital Pediatrics - 1.

The Muse Annexin V & Dead Cell Kit is a method to analyze cells in various stages of apoptosis, where fluorescently labeled Annexin V binds to phosphatidylserine (PS), which translocates to the outer surface of the cell membrane upon the onset of apoptosis. 7-AAD is excluded from live and healthy cells and permeates late-stage apoptotic and dead cells. In the untreated control, the live cells were 87.25%, which reduced to 74.40% when stimulated with Pg-LPS. However, upon treating Pg-LPS stimulated THP-1 cells with β-defensin 1, the live cells increased to 77.35%. This indicates that β-defensin 1 provided a protective effect to THP-1 cells. Additionally, Pg-LPS showed a necrotic effect, which was 22.85%. The result is depicted in Figure 9.

Hemolytic property of β-defensin 1

Using the HemoPred web tool, the hemolytic nature of β-defensin 1 was predicted. β-defensin 1 was found to be hemolytic in nature. 

## Discussion

One of the primary virulence factors of periodontitis, Pg-LPS, targets TLR4, which activates the NF-kB signaling pathway and triggers the secretion of inflammatory cytokines such as TNFα, IL-6, and chemokines, recruiting monocytes/macrophages to the site of infection [16]. AMPs not only possess antimicrobial activity but also exert anti-inflammatory properties. An antimicrobial peptide, cathelicidin, shows a potent LPS-neutralizing effect by directly interacting with LPS and reducing LPS-induced apoptosis in endothelial cells [17]. Cecropin A, a novel 37-residue cecropin-like antimicrobial peptide isolated from a cecropia moth, showed anti-inflammatory activities in mouse macrophage-derived RAW264.7 cells stimulated with LPS [18]. The synthetic tetrameric antimicrobial peptide, SET-M33D, synthesized from D amino acids, demonstrated broad-spectrum efficacy against both gram-positive and gram-negative bacteria, and it also had anti-inflammatory effects, decreasing the expression of inflammatory markers such as TNFα, IL-6, and COX-2 [19].

Two important functions of macrophages are to eliminate pathogens through phagocytosis and apoptosis of inflammatory cells and their subsequent clearance (efferocytosis). The present study analyzed the role of AMP, β-defensin 1, in Pg-LPS-stimulated THP-1 differentiated macrophage cells. In a molecular docking study carried out to understand whether β-defensin 1 interacted with Pg-LPS, it was found that β-defensin 1 had a good binding affinity with Pg-LPS [20]. Pg-LPS exhibited dose-dependent cytotoxicity in THP-1 cells. β-Defensin 1 showed dose-dependent cytotoxicity in THP-1 cells, and when Pg-LPS stimulated THP-1 cells were treated with β-defensin 1, cell viability increased up to 1 µg/ml of Pg-LPS, indicating the protective nature of β-defensin 1. It has been shown in a previous study that Pg-LPS suppresses phagocytosis via its effect on the cytoskeletal network [21]. The cytoskeleton is an integral component of the phagocytic process of macrophages. Pg adheres to the host cell surface, followed by internalization via lipid rafts and incorporation of the bacterium into early phagosomes [22]. LPS binds to β-tubulin and affects the F-actin-G-Actin equilibrium and inhibits microtubule and microfilament polymerization, altering cytoskeletal organization and thereby suppressing phagocytic function [21]. Bacterial exit is dependent on alterations in actin polymerization, lipid rafts, and microtubule assembly [23]. Pg-LPS activates phosphoinositide 3-kinase (PI3K), which prevents phagocytosis through the inhibition of RhoA GTPase and actin polymerization [24]. The PI3K/Akt pathway can modulate Rho GTPase activity by phosphorylating and inhibiting Rho GTPase-activating proteins (RhoGAPs). RhoGAPs normally promote the inactivation of Rho GTPases by accelerating their intrinsic GTP hydrolysis. By inhibiting RhoGAPs, the PI3K/Akt pathway can indirectly enhance Rho GTPase activity, leading to increased actin cytoskeleton remodeling. The results of the present study indicate that β-defensin 1 is able to protect the THP-1 differentiated macrophage cell line from the cytotoxic effects of Pg-LPS.

This study also investigated the anti-inflammatory potential of β-defensin 1, which showed that β-defensin 1 was able to inhibit the activities of COX, LOX, MPO, and inducible nitric oxide synthase. Pg-LPS infection of THP-1 cells resulted in an increase in ROS production, which decreased when treated with β-defensin 1, indicating the anti-oxidative activity of β-defensin 1. To determine whether β-defensin 1 affected the cell cycle of Pg-LPS-stimulated THP-1 cells, the cell cycle distribution was evaluated by Flow Cytometry. Administration of Pg-LPS increased the cells in the G0/G1 phase of the cell cycle, which was found to be reduced when co-treated with β-defensin 1. The apoptosis profile was studied by Flow Cytometry, and the results of the present study showed that when THP-1 cells were exposed to Pg-LPS, the percentage of live cells reduced when compared to the untreated control. However, when Pg-LPS stimulated THP-1 cells were treated with β-defensin 1, the percentage of live cells increased, showing a protective effect of β-defensin 1.

AMPs have the potential to exert their antimicrobial effects with less harm to normal eukaryotic cells due to the positive charge (cationic nature) on the surface of AMPs that can interact with the negatively charged membranes of microorganisms, while the eukaryotic cells are composed of uncharged neutral phospholipids, sphingomyelins, and cholesterol on the membrane surface [9]. Hemolysis refers to the rupture or destruction of red blood cells (RBCs) and can be a concern when working with peptides. β-Defensin 1 was predicted to be hemolytic in nature by the HemoPred web tool. Modifying the peptide sequence or structure can help reduce its hemolytic properties. This can be achieved by altering specific amino acid sequences or introducing substitutions such as replacing hydrophobic residues with hydrophilic ones. Amino acids such as lysine and glycine exhibit strong interaction with anionic bacterial membranes while having weak interactions with the zwitterionic phospholipid membrane of RBCs.

Hence, β-defensin 1 can serve as a multifunctional AMP, exerting a broad range of biological activities as evidenced by this present study. The AMPs can be synthesized and used therapeutically in topical applications, mouthwash, or toothpaste, and this, in turn, can reduce the severity of periodontal disease.

Limitations

The hemolytic nature of β-defensin 1 was predicted using the HemoPred web tool, and experimental analysis needs to be conducted to confirm its nature. The safety of β-defensin 1 should be tested in periodontitis-induced animal models for efficient treatment for periodontitis and associated inflammatory conditions.

## Conclusions

The results of the present study indicated that β-defensin 1 exerted a protective nature against the Pg-LPS stimulated THP-1 cell line. β-Defensin 1 can function as an anti-inflammatory and anti-oxidative cationic AMP. Cell cycle analysis revealed that β-defensin 1 reduced the Pg-LPS induced cell cycle arrest at the G0/G1 phase. Based on the apoptosis profile, β-defensin 1 increased the live cells when compared to THP-1 cells stimulated only with Pg-LPS. β-Defensin 1 may provide an alternate approach for fighting against periodontal infection and could offer a new therapeutic strategy for treating infections and inflammation-related illnesses.
